# Environmental and demographic risk factors for campylobacteriosis: do various geographical scales tell the same story?

**DOI:** 10.1186/1471-2334-12-318

**Published:** 2012-11-22

**Authors:** Julie Arsenault, Olaf Berke, Pascal Michel, André Ravel, Pierre Gosselin

**Affiliations:** 1 Faculté de médecine vétérinaire, Université de Montréal, Saint-Hyacinthe, Québec, Canada; 2 Laboratoire de lutte contre les zoonoses d’origine alimentaire, Agence de la santé publique du Canada, Saint-Hyacinthe, Québec, Canada; 3 Department of Population Medicine, University of Guelph, Guelph, Ontario, Canada; 4 Institut national de santé publique du Québec (INSPQ), Beauport, Québec, Canada, Centre hospitalier universitaire de Québec (CHUQ), Sainte-Foy, Québec, Canada; 5 Groupe de recherche en épidémiologie des zoonoses et santé publique, Faculté de médecine vétérinaire, Saint-Hyacinthe, Québec, Canada

**Keywords:** Campylobacteriosis, Quebec, Spatial, Poultry, Ruminants, Climate, Modifiable areal unit problem

## Abstract

**Background:**

Campylobacter is a common cause of bacterial gastro-enteritis characterized by multiple environmental sources and transmission pathways. Ecological studies can be used to reveal important regional characteristics linked to campylobacteriosis risk, but their results can be influenced by the choice of geographical units of analysis. This study was undertaken to compare the associations between the incidence of campylobacteriosis in Quebec, Canada and various environmental characteristics using seven different sets of geographical units.

**Methods:**

For each set of geographical unit, a conditional autoregressive model was used to model the incidence of reported cases of campylobacteriosis according to environmental (poultry density, ruminant density, slaughterhouse presence, temperature, and precipitation) and demographic (population density, level of education) characteristics. Models were compared in terms of number of significant predictors, differences in direction and magnitude of predictors, and fit of the models.

**Results:**

In general, the number of significant predictors was reduced as the aggregation level increased. More aggregated scales tend to show larger but less precise estimates for all variables, with the exception of slaughterhouse presence. Regional characteristics associated with an increased regional risk of campylobacteriosis, for at least some geographical units, were high ruminant density, high poultry density, high population density, and presence of a large poultry slaughterhouse, whereas a reduction in risk was associated with a lower percentage of people with diplomas, a lower level of precipitation, and warmer temperature. Two clusters of elevated residual risk were observed, with different location and size depending on the geographical unit used.

**Conclusions:**

Overall, our results suggest that the use of municipality or census consolidated subdivision were the most optimal scales for studying environmental determinants of campylobacteriosis at a regional level. This study highlights the need for careful selection and analysis of geographical units when using ecological study designs.

## Background

In Canada, as in many industrialized countries, infection with Campylobacter spp. is a leading cause of bacterial gastro-enteritis, with an annual average of 39 cases reported per 100,000 people over the last decade [1]. Many case–control studies have been conducted to identify risk factors for campylobacteriosis and have consistently revealed that some factors, including foreign travel, consumption of raw milk, eating in a restaurant, barbecuing, and coming into contact with raw poultry meat are associated with higher risk of disease [2]. Consumption of contaminated food is believed to explain approximately half of the reported cases [3-5]. In the last decade, environmental hypotheses have been put forward to explain campylobacteriosis risks that are not directly attributed to food [5,6]. Wild birds, poultry, sheep and cattle seem to be of particular importance in the natural life cycle of the bacteria, because they are easily infected and can excrete the bacteria in high numbers into the environment [7-14]. Aquatic environments are often contaminated by Campylobacter, which could make recreational and drinking water significant pathways of transmission between animals and humans [15-17]. Domestic flies could act as mechanical vectors for the transmission of the bacteria to humans as well [5]. Finally, meteorological factors likely affect the survival of the bacteria, and have been reported to influence the risk of disease [6,18-20].

Because of the regional-level intrinsic influence of environmental characteristics, the use of an ecological study design (i.e. the unit of analysis is a region) is a valid choice when studying these factors in relation to campylobacteriosis occurrence in populations. In the past, few ecological studies had been conducted to identify regional factors associated with the incidence of human campylobacteriosis. In Sweden, a positive association was found between the incidence of campylobacteriosis and both ruminant density and drinking water quality measures [17]. In the United States, counties with high poultry densities were reported to have higher incidence rates of campylobacteriosis [21,22]. In the province of Manitoba, Canada, the incidence of campylobacteriosis was reported to be highest among populations living in areas with high densities of farm animals, including cows, pigs, and chickens [23]. Although ecological studies such as these may reveal important characteristics linked to campylobacteriosis incidence, their conclusions may be influenced by the geographical scales used in the analysis, an issue known as the “modifiable areal unit problem” [24,25]. Results may also be biased if the boundaries of the geographical units of analysis do not follow the ones at which the process under study operates, an issue known as the zoning effect [24]. Although theoretical and empirical works have been published to better understand these design issues [25-29], we are not aware of any study directly addressing these issues for infectious diseases with environmental reservoirs. Exploring the impact of geographical scale on epidemiological inferences is also a valuable approach to better identify the various scales involved in the process [30].

The objective of this study was to estimate the effect of selected environmental and demographic characteristics on the regional incidence of human campylobacteriosis in Quebec using different geographical segregations of the study area, and to assess the impact of the choice of geographical units on epidemiological inference.

## Methods

An ecological study was conducted using human cases of campylobacteriosis reported in the province of Quebec, Canada, between 1996 and 2006 inclusively. The area under study was defined as the populated areas of the province, with the exclusion of non-organized territories, incompletely enumerated Indian reserves and settlements, and northern areas (Nunavik). Populated areas were defined as regions covered by census blocks in which at least one person was living according to the 2001 census of Statistics Canada.

### Data acquisition on campylobacteriosis cases

Following approval of the research protocol by the research ethics board of the Faculty of Medicine at the University of Montreal and by the research ethics board of the Agency of Health and Social Services of Montreal, available data on all laboratory-confirmed cases of Campylobacter infections reported in the province of Quebec for the study period were obtained from local health authorities. Cases were geocoded to the full 6-digit postal code areas, municipalities, and local community service center (CLSC). Recurrent cases occurring within less than 5 years of the first episode were excluded [31].

### Selection of geographical frameworks and scales

For the purpose of our study, we defined a geographical framework as a set of boundaries delineating an administrative or natural organization of the study area. These frameworks may include different subsets at various scales, which relates to the “aggregation effect” of the modifiable areal unit problem [24]. The different areas defined by the boundary of a geographical framework at a defined scale were termed geographical units, and refer to the notion of “zoning” effects [24]. Geographical sets of units used in this study are presented and defined in Table 1. These units were chosen among those previously described as applicable and of potential interest for the ecological study of campylobacteriosis in Quebec [32].

**Table 1:** Geographical units

**Geographical unit**^**a**^	**Number of units**^**b**^	**Description and sources of geographical data**
*Administrative*		
Municipalities	1,063	Municipalities (as determined by provincial legislation) or an area that is deemed to be equivalent to a municipality for statistical reporting purposes (e.g. cities, cantons). Source: Statistics Canada, 2006.
Census consolidated subdivisions	903	Grouping of adjacent census subdivisions. Generally the smaller, more urban census subdivisions (towns, villages, etc.) are combined with the surrounding, larger, more rural census subdivision in order to create a geographic level between the census subdivision and the census division. Source: Statistics Canada, 2006.
Census divisions	97	Groups of neighboring municipalities joined together for the purposes of regional planning and managing common services (such as police or ambulance services). Source: Statistics Canada, 2006.
*Health services*		
CLSC	155	Local community service center (CLSC) districts, which are the smallest health-related geographical division in Quebec. CLSC has the mission to provide local front-line health and social services to their population. Source: Quebec’s ministry of health and social services, 2004.
*Natural*		
Watershed	71	Drainage area boundaries at the sub-sub-basin level based on classic drainage basins having certain minimum volume of mean annual discharge. Source: Government of Canada, Natural Resources Canada, Canada Centre for Remote Sensing, The Atlas of Canada, 2007. Boundaries of watersheds were adjusted to fit the boundaries of the municipalities to avoid misalignment between population and covariate data (see [32] for more details).
*Custom*		
Smallest	1,119	Units equivalent to municipality or CLSC depending on which is the smaller.
Agriculture	319	Aggregated adjacent units from the smallest framework based on similar covariate patterns for presence of poultry production, presence of ruminant agricultural production and use of pasture (see [32] for more details).

Geographical units for the study of campylobacteriosis spatial distribution in Quebec, Canada.

^a^ Geographical units were hierarchical within the same framework (i.e. administrative, health services and custom), but not between frameworks.

^b^ Number of units are for the studied area.

### Definition and acquisition of environmental and demographic variables

Demographic and environmental variables were selected based on current literature and availability of data. They are defined in Table 2. Data on animal productions for the years 1998, 2001 and 2004 were obtained for each registered farm from the Ministry of Agriculture, Fisheries and Food of Quebec. Data were geocoded at the centroid of the main production site. For the calculation of ruminant density, only farms with enough ruminants to be registered—estimated to be 15 for small ruminants and 10 for bovine herds—were considered. The same rule was applied to the poultry density variable, with a minimum of 57 to 2100 birds required on the farms (depending on the type of flock) for inclusion. The total number of ruminants and poultry were then averaged over the 3 years of data using the weights 4, 3, and 4, respectively, before calculating densities by km^2^ of populated areas.

**Table 2:** Definition of risk factors

**Risk factor**	**Definition**
*Agricultural characteristics*	
Ruminant density per km^2^	Density of ruminants (goats, sheep, dairy cattle or beef cattle) per km^2^ of populated area.
Poultry density per km^2^	Density of poultry (hens, broilers, or turkeys) per km^2^ of populated area.
Slaughterhouse	Presence of ≥ 1 slaughterhouse handling poultry, cattle, and/or pigs under governmental inspection.
*Demographic variables*	
Diploma (%)	Percentage of people >15 years with a grade, certificate, or diploma.
Population density per km^2^	Total number of people living in the area out of the total area in km^2^.
*Climate variables*	
Precipitation (mm)	Average of daily precipitation for the study period.
Temperature (°C )	Average of the maximal and minimal daily temperature for the study period.

Risk factors tested for their association with campylobacteriosis in Quebec, Canada.

Slaughterhouse data for the year 2006 were obtained from the websites of the Canadian Food Inspection Agency and the Ministry of Agriculture, Fisheries and Food of Quebec. Slaughterhouses were geocoded using their civic number and street address within GeoPinPoint 2008 software [33]; manual geocoding at the street level was done whenever the automatic procedure was unsuccessful.

Demographic information was obtained for the census years 1996, 2001, and 2006 from Statistics Canada at the Dissemination Area level (i.e. smallest hierarchical division of the territory at which socio-economic data are released, comprising an average of 400 people). For each census year, data from each Dissemination Area within a geographical unit were averaged, weighted by their population size. For each geographical unit, data were then averaged over the 3 census years using weights of 2.5, 5, and 2.5, respectively.

Climate variables were obtained from the National Land and Water Information Service of Agriculture and Agri-Food Canada for 1996 to 2003, inclusively. These values represent a 10x10 km cell size, generated by the interpolation (ANUSPLIN v4 algorithm) of meteorological data collected from weather stations across Canada. The rasters were first transformed at a cell size of 0.5 km using the Spatial Analyst extension of ArcInfo 9.2 [34], and then average values of raster cells within each geographical unit were computed. This was necessary as some smaller units did not cover any pixel center of the original raster, creating missing values when extracting data.

### Statistical modeling

All covariates were categorized prior to modeling in order to get a constant set of predictors for all models and/or because the variable to outcome relationship was assumed to be non-linear in form. Animal density variables were put into 3 categories: absence, medium levels, and high levels of production. The cut-off for high levels was set at the 85th percentile of the respective empirical distribution at the level of municipalities. The slaughterhouse variable was organized into 3 categories: absence of any kind of slaughterhouse, presence of large poultry slaughterhouse(s), and presence of small poultry slaughterhouse(s) or slaughterhouse(s) for other species. A poultry slaughterhouse belonging to one of the four largest poultry transformation companies in Quebec, which have a combined market share of over 90%, was defined as “large” [35]. For demographic and climatic variables, 3 categories were defined using the 15th and 85th percentile of their empirical distributions at the level of municipalities.

For statistical modeling, the dependent variable was defined as the average number of reported cases of campylobacteriosis per 100,000 people per year. The incidence was directly standardized for age group (0–4 yrs, 5–15 yrs, 16–44 yrs, >45 yrs), using the study population for the year 2001 as the standard population. Prior to modeling, the incidence rate was smoothed using the empirical Bayesian estimation in order to improve normality and variance homogeneity [36]. This procedure also has the advantage of avoiding the use of transformed scales, which can be difficult to interpret [37]. Ordinary regression models were built for each geographical unit without consideration of the spatial structure of the data. All covariates were included as fixed effects, with no interactions. No variable selection was done in order to avoid any influence of the selection method or covariate(s) exclusion in the comparison of models. Models were built using SAS software [38]. Studentized residuals were computed from the final models to assess the spatial dependence. An empirical semi-variogram was estimated based on Euclidean distances, with a 95% confidence band estimated in R (package geoR [39]). The choice of Euclidean distances to evaluate the spatial dependence in residuals was based on preliminary analyses (see Additional file 1).

In the presence of spatial structure in studentized residuals from the ordinary regression models, a conditional autoregressive (CAR) model was fit in R using the “spdep” package [40] for each set of geographical units. A binary neighbor matrix was used to account for spatial autocorrelation. The cut-off within which surrounding areas were considered neighbors was determined as the practical range of the semi-variogram using studentized residuals from ordinary regression models. The practical range was estimated by maximum likelihood in R using the package “geoR” [39], based on an exponential model. All variables were included as fixed effects. Residuals from the final CAR models were visually assessed for normality using QQ-plots.

The geographical sets of units were first compared based on the estimated associations between outcome and exposure variables. More specifically, the number of exposure variables with statistically significant associations was compared between sets of units. Among significant variables, differences in direction and magnitude in risk estimates were also described. For the comparisons in terms of magnitude, the maximal percentage of variation between statistically significant coefficient estimates for each explanatory variable was calculated as follows:

$$\left|\left({\hat{\beta }}_{\max}-{\hat{\beta }}_{\min}\right)÷{\hat{\beta }}_{\min}\right|\times 100)$$

The geographical sets of units were then compared in term of model fitting. Three criteria were used: 1) Pearson’s coefficient of correlation (r^2^) between observed and predicted outcome (a good fit is indicated by strong correlation); 2) spatial dependence in model residuals as measured by Moran’s I using the same neighborhood definition as used in the conditional autoregressive models; and 3) presence of areas of unexplained higher risk. This was evaluated with a spatial scan test scanning for primary and secondary clusters of high residual values based on the normal model, performed in SaTScan version 8.1.1. [41]. Significant clusters (p<0.05) were mapped.

## Results

A total of 28,521 cases of campylobacteriosis reported in the study area between 1996 and 2006 were included in the analysis. The average annual population within the study area was 7,367,517 people, giving an overall estimate of annual incidence of campylobacteriosis of 35.2 cases per 100,000 people. The size of the study area was 218 668 km^2^.

The distribution of exposure variables included in regression models is presented in Table 3. For all ordinary regression models, there was evidence of spatial dependence among studentized residuals. Thus, CAR models were built for all sets of geographical units. Results from CAR models are presented in Table 4. Neighbors were defined as units with population centers within the practical range estimated from the semi-variogram, ranging from 15 km to 34 km depending on the model (see Table 5). Residuals from all CAR models were normally distributed according to visual inspection.

**Table 3:** Distribution of risk factors

	**Smallest**	**Muni-cipality**	**Census consolidated subdvision**	**Agri-culture**	**CLSC**	**Census division**	**Water-shed**
	**(n=1119)**	**(n=1063)**	**(n=903)**	**(n=319)**	**(n=155)**	**(n=97)**	**(n=71)**
Ruminant density per km^2^							
>20	204	204	179	64	10	9	1
≤20	736	721	661	184	97	85	59
None	179	138	63	71	48	3	11
Poultry density per km^2^							
>250	185	185	169	46	28	29	12
≤250	132	128	128	54	48	46	28
None	802	750	606	219	79	22	31
Slaughterhouse^a^							
Large poultry	6	6	6	5	5	5	5
Others	41	41	41	25	32	31	16
None	1072	1016	856	289	118	61	50
Diploma (%)							
<50	209	208	192	37	5	3	14
50-75	724	701	615	209	96	74	51
>75	186	154	96	73	54	20	6
Population density per km^2^							
≤6	207	206	193	20	n/a^b^	n/a^b^	7
6-400	683	681	589	191	47	46	32
≥400	229	176	121	108	108	51	32
Precipitation (mm)							
<2.9	138	136	121	40	13	10	13
2.9-3.1	775	729	607	214	117	69	50
>3.1	206	198	175	65	25	18	8
Temperature (°C)							
<3.1	191	189	177	55	24	20	32
3.1-6.9	755	739	639	220	79	66	36
>6.9	173	135	87	44	52	11	3

Distribution of risk factor values for each spatial set of units.

^a^ A total of 52 slaughterhouses were present, handling poultry (n=24), ruminants (n=17) and/or swine (n=23). The “Others” category refers to areas having slaughterhouses handling ruminants or swine and might also include poultry slaughterhouses.

^b^ Not applicable (absence of areas with those values for the specific unit set).

**Table 4:** Regression coefficients of CAR models

	**Geographical unit**							**Maximal % of variation**
	**Smallest**	**Municipality**	**Census consolidated subdvision**	**Agriculture**	**CLSC**	**Census division**	**Watershed**	
**Variable**	**(n=1119)**	**(n=1063)**	**(n=903)**	**(n=319)**	**(n=155)**	**(n=97)**	**(n=71)**	
Intercept	34.32 (1.83)*	35.98 (2.11)*	35.12 (2.37)*	39.54 (2.80)*	36.38 (3.97)*	23.35 (8.79)*	16.33 (6.34)*	
Ruminant density per km^2^ (ref.=none)								
>20	8.72 (2.04)*	7.17 (2.14)*	10.76 (2.57)*	4.47 (3.21)	4.75 (5.20)	17.37 (10.55)	16.00 (13.10)	50
≤20	5.44 (1.63)*	3.25 (1.76)	4.68 (2.21)*	3.21 (2.40)	2.96 (3.41)	15.05 (8.98)	8.88 (5.77)	16
Poultry density per km^2^ (ref.=none)								
>250	3.44 (1.52)*	2.06 (1.53)	3.84 (1.50)*	−1.32 (2.75)	1.24 (3.82)	4.17 (4.69)	19.02 (7.55)*	453
≤250	−0.28 (1.62)	−1.05 (1.60)	1.68 (1.48)	−4.62 (2.48)	−1.15 (3.28)	1.36 (4.50)	18.38 (4.59)*	
Slaughterhouse (ref.=none)								
Large poultry	65.90 (6.75)*	59.24 (6.47)*	27.81 (5.91)*	24.68 (7.33)*	20.42 (5.91)*	15.99 (6.84)*	1.96 (7.14)	312
Others	−1.18 (2.63)	0.94 (2.53)	−1.40 (2.30)	−2.51 (3.33)	1.72 (2.96)	1.52 (3.41)	−5.74 (4.58)	
Diploma in % (ref.=50-75)								
<50	−3.80 (1.42)*	−3.76 (1.41)*	−4.25 (1.34)*	−4.71 (3.09)	−18.16 (6.49)*	−19.68 (9.06)*	−6.37 (4.38)	423
>75	−0.20 (1.54)	−1.98 (1.64)	−2.04 (1.78)	−1.72 (2.50)	−4.58 (2.56)	−6.81 (4.13)	5.24 (6.00)	
Population density per km^2^ (ref.=6-400)								
≤6	−2.25 (1.47)	−2.48 (1.49)	−1.28 (1.38)	−6.78 (4.09)	n/a^b^	n/a	3.64 (5.66)	
>400	3.58 (1.50)*	4.59 (1.52)*	0.36 (1.53)	5.69 (2.16)*	0.91 (2.76)	2.93 (3.21)	0.68 (3.75)	59
Precipitation in mm (ref.=2.9-3.1)								
<2.9	−3.91 (1.86)*	−3.60 (2.40)	−4.56 (2.07)*	−9.59 (3.49)*	−14.09 (4.52)*	−15.96 (5.39)*	−7.92 (4.51)	308
>3.1	1.33 (1.57)	1.09 (1.99)	1.19 (1.81)	3.18 (2.94)	8.89 (3.55)*	5.80 (4.31)	5.61 (5.35)	
Temperature in °C (ref.=3.1-6.9)								
<3.1	1.08 (1.65)	−3.45 (2.18)	−1.55 (1.88)	1.67 (3.08)	3.14 (3.39)	7.03 (3.90)	10.25 (4.05)*	
>6.9	−9.50 (1.92)*	−4.00 (2.57)	−5.32 (2.61)*	−10.42 (3.42)*	−6.47 (3.87)	−6.43 (5.06)	−17.30 (8.73)*	225
Lambda^c^	0.58*	0.8*	0.68*	0.45*	0.59	0.56*	0.31*	
Model r^2^	0.21	0.27	0.26	0.24	0.48	0.37	0.52	

Regression coefficients (standard errors) of conditional autoregressive models predicting the annual incidence of campylobacteriosis per 100,000 people in Quebec, Canada, 1996–2006.

^a^ Maximal percentage of variation in statistically significant estimates of regression coefficient based on different geographical units.

^b^ Not applicable (absence of areas with those values for the specific unit set).

^c^ Spatial error parameter adjusting for spatial correlation.

*p<0.05.

**Table 5:** Cluster and clustering in residuals

	**Smallest**	**Munici-pality**	**Census consolidated subdivision**	**Agri-culture**	**CLSC**	**Census division**	**Water-shed**
	**(n=1119)**	**(n=1063)**	**(n=903)**	**(n=319)**	**(n=155)**	**(n=97)**	**(n=71)**
*Cluster (scan test)*							
Principal cluster							
Mean^a^ inside	79.97	74.57	77.99	None	10.37	12.35	None
Mean^a^ outside	−0.22	−0.14	−0.091		−2.49	−2.6	
Number of units	3	3	2		29	17	
Area (km^2^)	219	219	203		42993	40052	
Location^c^	A	A	A		B	B	
P-value	<0.01	<0.01	0.04		<0.01	0.02	
*Clustering*							
Moran’s							
Neighbors (km)^b^	27	29	19	30	18	15	34
Estimate	−0.06	−0.02	−0.11	−0.06	−0.02	0.09	0.02
P-value	0.99	0.98	0.99	0.90	0.63	0.34	0.42

Regression coefficients (standard errors) of conditional autoregressive models predicting the annual incidence of campylobacteriosis per 100,000 people in Quebec, Canada, 1996–2006.

^a^ Mean of residuals.

^b^ Pairs of units with population centers within this distance were considered neighbors.

**Figure 1:**
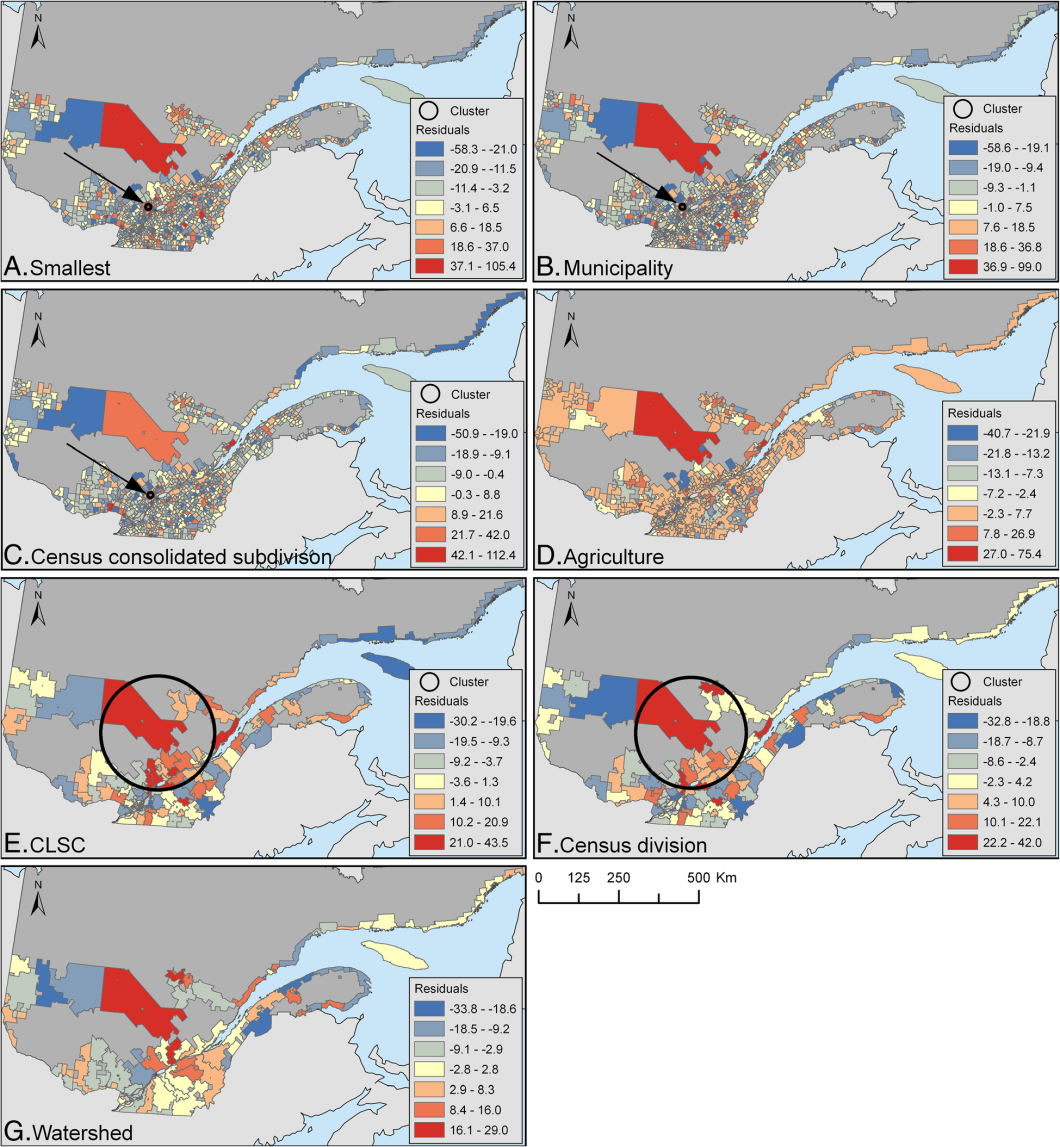
**A-G. Distribution of residuals.** Residuals from a CAR model predicting the annual incidence of reported cases of campylobacteriosis per 100,000 people in Quebec (1996–2006) according to various geographical units. Significant clusters (p<0.05) in residuals according to the scan test are illustrated. Classification was done using Jenk’s natural breaks. Dark grey areas represent the unpopulated areas, non-organized territories or incompletely enumerated Indian reserves and settlements within Quebec, whereas light grey shows the frontier area of Quebec.

^c^ A similar letter indicates a similar location for the cluster. See Figure 1 for an indication of the exact cluster location.

The number of exposure variables significantly associated with campylobacteriosis incidence in CAR models tended to decrease as the level of aggregation increased (Table 4). For all variables, the direction of the association for statistically significant estimates was consistent across all sets of geographical units. A large variability was detected in point estimates of statistically significant variables obtained from different geographical units, ranging from 16% to 453% (Table 4). In general, the estimates of regression coefficients increased with a high level of aggregation, with the exception of slaughterhouses for which a reverse trend was noted. The “poultry density” was particularly variable, and this high degree of variation was driven by the coefficients estimated at the watershed level, which were three times higher compared to others.

**Figure 2:**
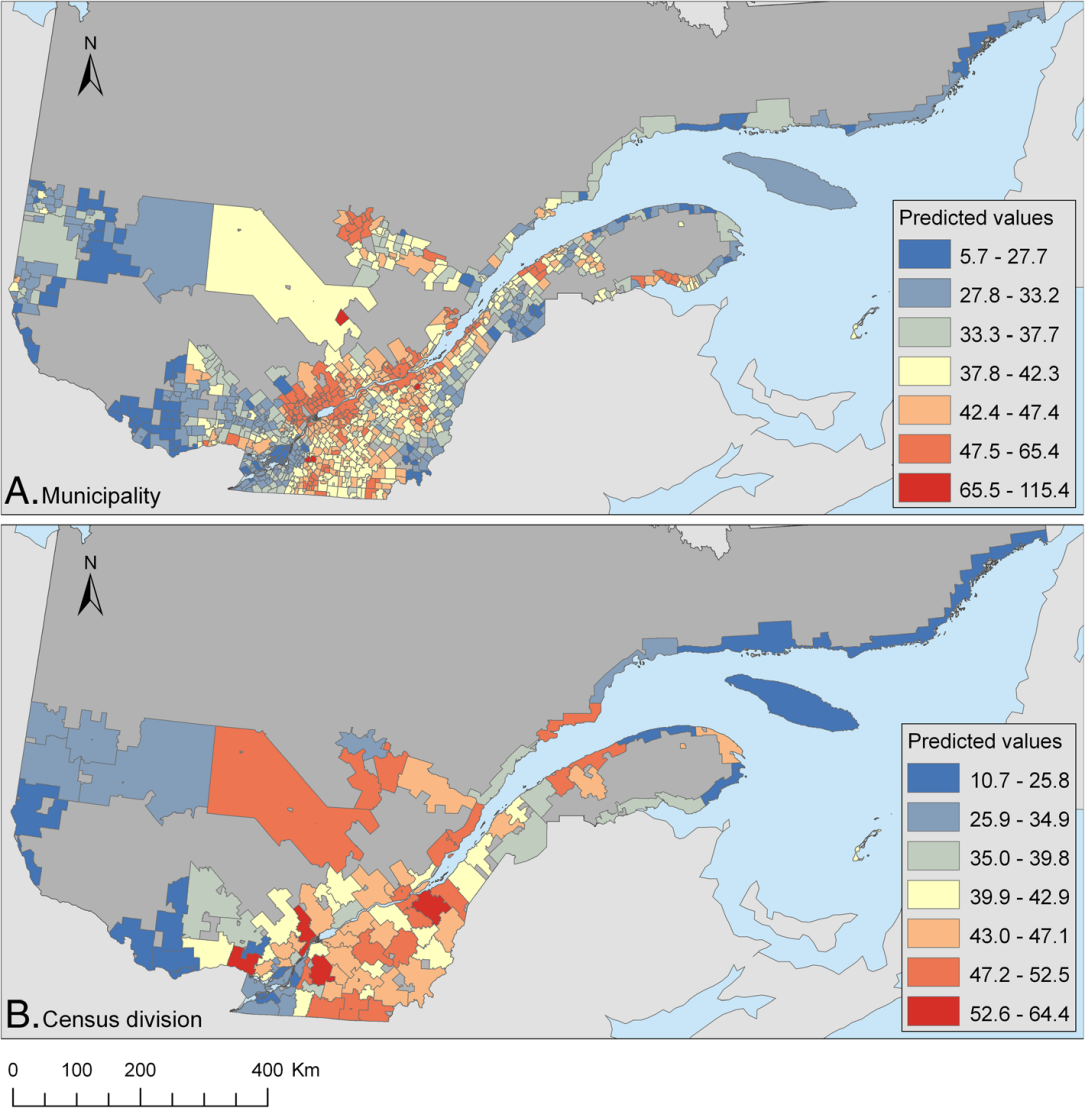
**A-B. Predicted incidence of campylobacteriosis.** Predicted annual incidence of reported cases of campylobacteriosis per 100,000 people in Quebec (1996–2006) according to a conditional autoregressive (CAR) model at the level of municipality (**A**) or census division (**B**). Classification was done using Jenk’s natural breaks. Dark grey areas represent the unpopulated areas, non-organized territories or incompletely enumerated Indian reserves and settlements within Quebec, whereas light grey shows the frontier area of Quebec.

The Pearson r^2^ between the observed and predicted values of the CAR models ranged from 21% to 52%, with an increasing trend as the data got more aggregated (Table 4). No clustering was detected in residuals according to Moran’s I test (Table 5). The scan test detected the same small hotspot area in residuals for the three sets of geographical units at a smaller scale, whereas a very large hotspot was identified for two of the geographical units at a larger scale (Table 5 and Figure 1). This large area was located in a zone with a high predicted incidence of campylobacteriosis (see Figure 2). No secondary cluster was found to be significant (all p>0.05).

## Discussion

The various geographical sets of units gave various insights into the spatial distribution of campylobacteriosis incidence. These differences can be attributed to a change in scale (e.g. for administrative units) or to a combination of changes in scale and zoning. Different criteria were used to compare the results obtained from different sets of geographical units, selected for their epidemiological relevance. First, the difference in estimated associations between campylobacteriosis incidence and exposure variables was explored, since different results can lead to a different understanding of the environmental factors influencing the spatial distribution of the disease. Next, residual clustering or hotspots of unexplained risk were explored. Local zones of unexplained risk were detected, which potentially identify different scales at which disease processes are occurring and specific areas and scales warranting further investigation.

### Associations between campylobacteriosis and exposure variables

The number of significant variables in the CAR models was reduced as the level of aggregation increased. This was likely driven by a reduction of statistical power due to lower sample size rather than reduced strength of association as the point estimate in regression coefficients were in general larger in more aggregated data. It should be noted that, with the exception of covariates referring to animal density, significant effects on regression coefficients were only observed for those covariates showing less variability at lower level geographical units [32]. Choice of unit based on minimal intra-zonal variance was suggested as a potential solution to the modifiable areal unit problem, as recently reviewed [25]. The agriculture-based custom framework was created in order to increase the size of units to improve stability in rates, while preserving high intra-unit homogeneity in animal production. However, it has the drawback of being heterogeneous in unit size and many units had a non-compact shape, which might have hampered the ability to find statistically significant associations.

The direction of the associations was consistent and in agreement with current biological knowledge. Poultry and ruminants are frequently colonized with Campylobacter and can shed the bacteria in high numbers [10,42-44]. Contamination in humans can then occur through direct contact with these animals or following indirect contact with a contaminated environment. We thus expected a positive association with a likely dose–response relationship, as we observed. For climate variables, the directionality of the association was also compatible with Campylobacter biology, which has reduced survival in a warmer environment and is sensitive to desiccation [45-49]. The inclusion of education as predictor variable was justified by a previous study reporting its influence on some steps needed for a case to be reported [50], with potential bias on spatial patterns. We observed a reduction in the incidence of campylobacteriosis with a lower level of education, which has also been reported by others and might represent a proxy for socio-economic status [51]. Traveling and eating in a restaurant were reported as risk factors for campylobacteriosis in other countries, which are also associated with socio-economic variables including the level of education [51,52]. To our knowledge, the importance of traveling as a risk factor for campylobacteriosis has not been evaluated in Canada, but is supported by a study conducted in a Canadian community, where about 22% of reported cases of campylobacteriosis were associated with international traveling [53]. We could not exclude travel-related cases from our study because this information was missing for most cases. The biological pathway linking slaughterhouse presence to campylobacteriosis incidence is twofold: professional exposure of poultry slaughterhouse workers was previously reported to increase the risk of campylobacteriosis [54]; and slaughterhouse effluents were reported to harbor large quantities of Campylobacter [55], which could then contaminate the surrounding drinking or recreational water. However, we do not have a clear explanation for positive association between campylobacteriosis incidence and population density observed for some geographical units, apart from some effects due to differences in case reporting.

The slaughterhouse variable was associated with campylobacteriosis incidence, with a significant decrease in magnitude with aggregation and non-overlapping confidence intervals between geographical units. This met our expectation for this particular variable, since its effect would likely be limited to the nearby areas where workers live or environmental contamination occurs, and a reduction of the coefficient in the aggregation process would occur due to a dilution effect. We further explored these associations by multiplying the point estimate of large poultry slaughterhouse coefficients with the total populations at risk living in areas with large poultry slaughterhouses. In summary, the case load due to the presence of large poultry slaughterhouses in the study area ranged from 9 to 172 cases, depending on the framework. This illustrates the impact of choice of geographical units on potential public health conclusions.

For all other variables, the estimated coefficients of regression tend to increase in magnitude as the units become more aggregated, but this increase was not consistent across all geographical sets of units and thus not predictable. This increase is in accordance with previous work based on simple linear regression models, although another study conducted in a multivariate regression setting reported that the results were essentially unpredictable [29]. For the animal density variables, the estimated regression coefficients with the highest values also had large standard errors, and were not statistically significant. Among the statistically significant ones, the variation in point estimates was too small in our perspective to affect epidemiological conclusions on the biological relevance of these variables. The only exception was perhaps for poultry density at the watershed level. At this level of aggregation, we suspected the presence of colinearity between poultry density and ruminant density based on our knowledge of the study area. Also, according to a simulation study, the aggregation process can introduce colinearity between exposure variables where none existed before [26]. In order to explore this possibility, alternative models were built by excluding either the poultry density or the ruminant density from the model. For the watershed units, the exclusion of the poultry density variable led to larger and significant coefficients for the ruminant density variable (from 16.0 [p=0.22] to 23.1 [p=0.08] for ruminant density >20 per km2 and from 8.9 [p=0.12] to 15.9 [p<0.01] for ruminant density ≤250 per km^2^). This is suggestive of colinearity between these two variables, and so the effect attributed to poultry or ruminant density for frameworks at a larger scale is probably a cumulative effect of both types of production. Indeed, any epidemiological study aiming to study the distinct effect of poultry and ruminant density should avoid any use of geographical units at a scale larger than census consolidated subdivisions. From another perspective, the large regression coefficients for animal densities observed at the watershed scale could be indicative of real biological processes occurring at that specific scale, which is not impossible considering the importance of aquatic environments in the ecology of Campylobacter [56].

For the demographic and climatic variables, the point estimates of regression coefficients also increased with aggregation, although confidence intervals increased and point estimates were not statistically different between geographical units (i.e. point estimates for one geographical unit were included in the confidence intervals of estimates from other geographical units). It should be noted that high values of estimated regression coefficients for climate variables as seen for the “smallest” and “CLSC” geographical units compared to “municipality” are probably misleading. In fact, 51 and 39 of the divisions constituting these units were located on the islands of Montreal and Laval, respectively, urban areas characterized by warm temperatures and low amounts of precipitation. At the scale where it was measured, almost no variation in weather variables was seen within this area; indeed, these areas were probably over-represented in the estimated relationship between campylobacteriosis and weather variables.

### Evaluation of the fit of models

The fit of models was explored in different ways. As previously reported, an increase of the correlation coefficient between observed and predicted data was seen as the level of aggregation increased. This is attributed to the smoothing effect of aggregation [25,29] and highlights the fact that too much emphasis should not be placed on the interpretation of this measure. The absence of clustering in residuals was suggestive of a good adjustment for spatial dependence in all models. And finally, a small hotspot of unexplained residual risk was detected by the scan test for the three sets of geographical units at the smallest scales, i.e. the “smallest,” “municipality,” and “census consolidated subdivision” geographical units. Considering the small size of this hotspot, it was likely smoothed out in the aggregation process for geographical units at a large scale. A community episode of campylobacteriosis from a common source is a potential explanation for this high risk zone, or it could have been caused by a locally-acting factor not measured in our study. Molecular typing data on Campylobacter strains were not available from our dataset, but would have been very useful in distinguishing between these two processes, as we expect a higher similarity of strains isolated within a cluster in the presence of an outbreak from a common source [57]. For two of the geographical units at a large scale (i.e. CLSC and census division), a large area of significant unexplained risk was detected. This area was characterized by high poultry production and relatively high predicted risk of campylobacteriosis. We do not have a clear explanation for this finding. It is possible that in areas of high animal density, the effect of environmental variables on campylobacteriosis risk is amplified in a non-linear way due to the dynamic of bacterial transmission between reservoirs. In more aggregated units, the areas included in the average animal density categories were less homogeneous, which might lead to poorer adjustment of the model. Geographically-weighted regression might be useful in exploring this possibility, but this was beyond the scope of our study.

### Limitations

The choice of framework and scales included in the study were limited to the ones at which cases could be located. Additionally, only cases reported through the surveillance system were included, limiting the generalization of results to all cases occurring in the population. In fact, case reporting involves multiple steps, which could be influenced by health care system, physician, or patient characteristics. In the province of Quebec, health care services are accessible to all, with universal health insurance plan allowing the entire population to receive free hospital and medical services, which should limit any related reporting bias. Regarding physicians, their propensity to ask for a stool sample have been reported to vary according to their speciality and severity of illness of their patients [58]. Also, the decision of a diarrheic patient to consult a physician has been reported to depend upon severity of illness, urbanicity, and various socio-economic indicators of patients [50,59]. Our models only offer partial adjustment related to patient characteristics, with the inclusion of an educational status variable. Thus, we cannot exclude that the observed association with environmental risk factors were biased by spatially-varying factors influencing steps conducting to the reporting of a case. However, as we used the same dataset for all geographical sets of units, the underreporting bias was kept constant and this should not invalidate our conclusions regarding the influence of geographical units on risk factor estimation. Also, our results could be dependent on the method used for statistical modeling. Using the Poisson regression model with spatially correlated random effects was explored for our study, but no convergence was obtained for most models. Our study design did not allow us to point out which mechanisms were exactly responsible for the differences observed. Theoretical studies on modifiable areal units assume that unit size does not vary much, but in our case we had small urban units with large rural units in most frameworks, and this could complicate interpretation.

## Conclusions

Our study allowed us to evaluate to what extent the modifiable areal unit problem influences the epidemiological conclusions on the spatial distribution of campylobacteriosis. In general, the various geographical units gave consistent results for the direction of the associations between incidence and various environmental and demographic characteristics, which were in agreement with current knowledge on campylobacteriosis epidemiology. However, the strength and statistical significance of the associations observed at one scale should be used with caution when inference is made at another scale. In general, municipality or census consolidated subdivision scales are recommended for the study of the spatial distribution of campylobacteriosis in our study area, based on theoretical grounds [32] and low degree of colinearity in agriculture variables at this scale. These were also associated with significant and biologically meaningful associations between campylobacteriosis incidence and the exposure variables under study.

## Competing interests

The authors declare that they have no competing interests.

## Authors’ contributions

JA designed the methodology, performed the statistical analyses and drafted the manuscript. OB supervised the analyses. PM, AR and PG assisted in designing the methodology and drafting the manuscript. All authors read and approved the final manuscript.

## Pre-publication history

The pre-publication history for this paper can be accessed here:

http://www.biomedcentral.com/1471-2334/12/318/prepub

## Supplementary Material

Additional file 1 Choice of distance matrix. Click here for file
