# Supplementary material for: Environmental and demographic risk factors for campylobacteriosis: do various geographical scales tell the same story?
Source: BMC Infect Dis. 2012 Nov 22;12:318. doi: 10.1186/1471-2334-12-318 (PMC3570353; doi:10.1186/1471-2334-12-318)
Supplement: Additional file 1 — Choice of distance matrix. [file 1471-2334-12-318-S1.doc]

# Additional file 1. Choice of distance matrix

The choice of the distance matrices for the adjustment of potential unexplained spatial dependence in regression models was selected based upon an exploratory analysis of the studentized residuals from the final ordinary regression models. Spatial dependence was evaluated using 3 different distance measures between geographical units: Euclidean distance, travel time (based on road speed limits), and travel distance . Calculation of distances was based on the population centroid of each geographical unit, defined as the geographical mean center of census tracts that form the area, and weighted by the population of those census tracts in 2001. All distance calculations were done on projected coordinates using a Lambert conic conform projection parameterized for our study area. For travel time and distances, calculations were performed with the Network Analyst extension of ArcInfo 9.3 using the CanMap Streetfiles network .

Spatial dependence was evaluated by estimating Moran’s I from studentized residuals, with p-values resulting from permutation tests performed in R with the “spdep” package . For the various distance measures, Moran’s I was estimated for distance bands of 15 minutes or 15 kilometer widths of up to half of the maximal distance, and correlograms were used to explore the results. For the Euclidian distance, an empirical semi-variogram was also used to assess the spatial dependence, with a 95 % confidence band estimated in R (package geoR ).

The studentized residuals from all final ordinary regression models strongly suggested the presence of spatial dependence at a distance of around ≤40 km or ≤40 minutes, as illustrated in Additional Figure 1 for municipalities. The only exceptions were for the watershed and CLSC frameworks, for which the correlogram weakly suggested the presence of spatial dependence in a similar range, but patterns were blurred by random variations (not shown). Considering that the various definitions of distance gave close sets of neighbors with similar spatial dependence, the Euclidian distance was selected to take into account the spatial dependency (if needed) for all sets of geographical units for consistency.

**Additional** **Figure 3 Correlogram of residuals.** Correlogram of residuals from a linear regression modeling the standardized annual incidence of campylobacteriosis in municipalities of Quebec, Canada, 1996–2006


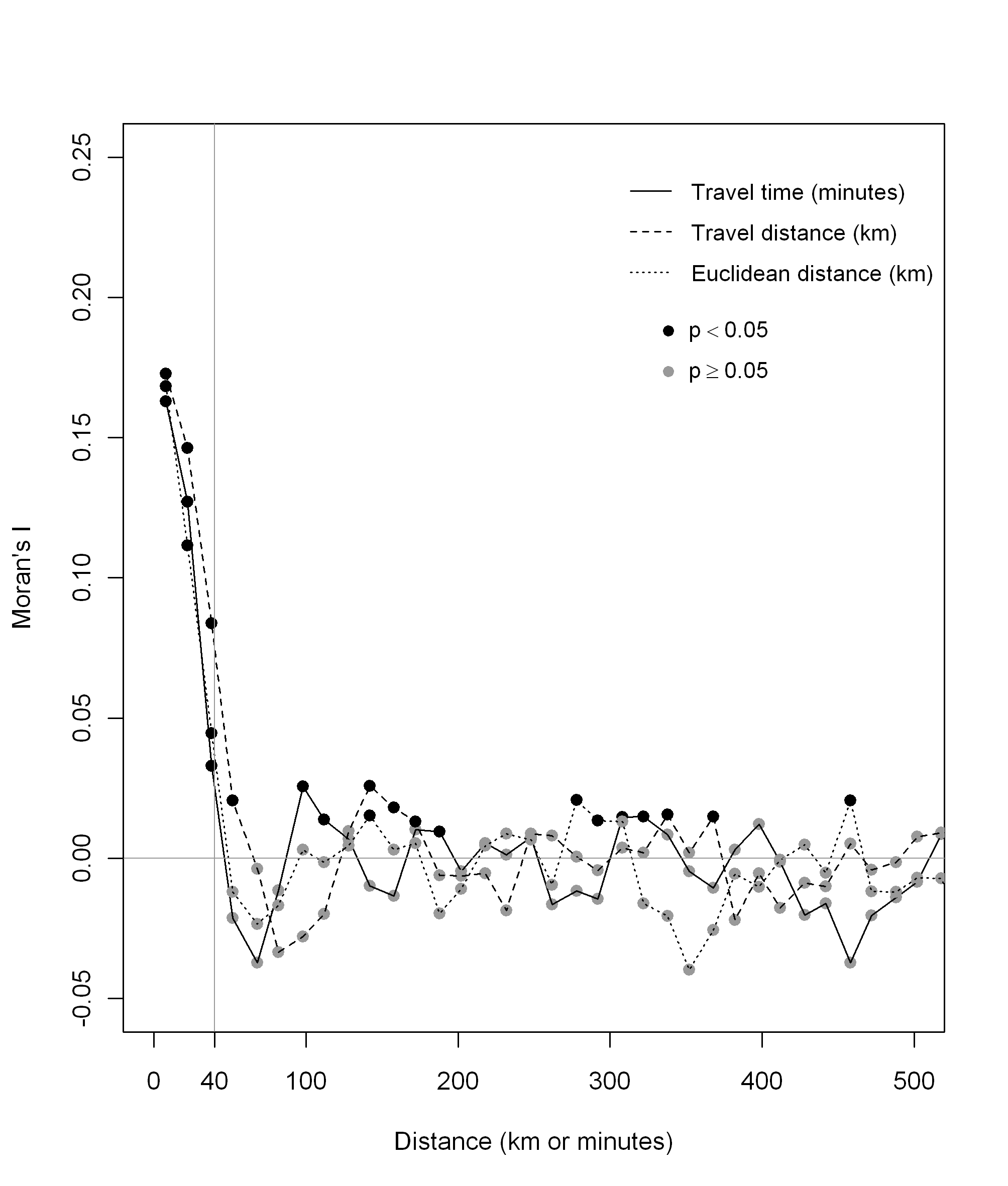


1. Mitchell A: **The ESRI Guide to GIS Analysis**, vol. 1. Geographic patterns & relationships. New York: ESRI Press; 1999.

2. Waller LA, Gotway CA: **Applied spatial statistics for public health data**. Hoboken, New Jersey: John Wiley & Sons Inc.; 2004.

3. DMTI Spatial Inc.: **CanMap Streetfiles version v2007.3**. In*.* Markham (Ontario), Canada; 2007.

4. Bivand R: **spdep: Spatial dependence: weighting schemes, statistics and models. R package version 0.5-4**; 2010.

5. Ribeiro PJJ, Diggle PJ: **geoR: a package for geostatistical analysis**. *R-NEWS* 2001, **1**(2):15-18.
